# Calomel and Tartar Emetic in the Army

**Published:** 1863-08

**Authors:** 


### CALOMEL AND TARTAR EMETIC IN THE ARMY.

Reprinted from the "American Medical Times."

*To the Editor of the American Medical Times:*

Sir:—Participating in that national sensibility which causes Americans to shrink from the touch of anything that may tarnish our national escutcheon, I was wounded by the circular of the Surgeon-General, dated May 4, 1863, which directed calomel and tartar emetic to be "struck" from the Medical Supply-Table of the army; and this the more deeply, because, from the official eminence of its source, it might be regarded on the other hemisphere as a proof of our professional retrogression. When contemplated from another point of view, whence it may be looked at as an evidence of our individual and national imbecility, as shown in the readiness with which we bend to every breeze of fanaticism, whether freighted with medical or political heresy, it is then sufficiently humiliating, and should be met with a rebuke from every conservative member of the profession.

The efforts of the *American Medical Times* to justify this act, and to sustain and sanctify this error by its influence over its readers, have induced me to make this reply, not more to the circular than to the remarks of its editor in vindication of it.

I can find excuses for the action of this officer in the fact that he has seen but little of military service, and knows but little from personal observation of the diseases of soldiers; and also in the influence imputed to the National Sanitary Commission over the Chief of the Medical Bureau, to the head of which commission, he being clothed with a sacredotal pallium, we may, perhaps, justly impute a full share in the introduction of this species of medical fanaticism into the army of the United States.

For the gentleman who made a speech in defence of the circular before the American Medical Association, at Chicago, an apology may be framed on the supposition that it was for his interest to do so. The official relation of the parties renders such a supposition quite probable, and would, in other courts, impeach his testimony.

Whilst in my own mind I can satisfactorily account for the action of the Surgeon-General, and apologize for the indiscretion of his friend in the Medical Association, I am entirely at a loss for an explanation of the course pursued by the editor of the *American Medical Times*, in bringing the influence of a journal heretofore aiming to be the exponent of the medical opinion of the country to the defence of an act offensive to that profession and insulting to the medical staff of the army. One reason given by the Surgeon-General for the erasure of calomel and tartar emetic from the army medical Supply-Table, viz., that it was done in consequence of the teachings of "modern pathology," may deceive the readers of "Physiological Essays," but cannot mislead the students of historic medicine, who know how much therapeutics are indebted to empiricism, in its ancient signification, for the introduction of many important articles into the *materia medica* prior to the time of Galen, and who are also aware that the usages of that sect who tested the utility of remedies by experimentation still furnish the means by which judgments are formed of their claims to a position in our works on therapeutics.

The other reason, judging from its position, the first in importance in the estimation of the Surgeon-General, rests upon reports of the Sanitary Inspectors, who have assumed that certain forms of humid gangrene seen among the troops in the United States service are the effects of the administration of mercury; and from thence the conclusion is arrived at, that the evils consequent upon its use more than counterbalance the good to be attained by its further toleration as a remedy, wherefore it is "struck" from the Supply-Table of the medical department of the army. That neither of the parties advocating the enforcement of the order excluding calomel from the army hospitals should have uttered a doubt as to the nature or the cause of the gangrene alluded to, is a painfully significant fact, showing either ignorance of the medical history of the country or a disposition to stifle its teaching.

If the facts assumed by the Surgeon-General to be true are not to be called in question, they only prove what all right-minded people admit, the predominance of evil in this world everywhere. All intelligent members of the medical profession know that the mischiefs done in the name of medicine outweigh the good it has accomplished. And what we admit to be true of medicine, we believe to be also true of law and divinity; but no sane person would on that account for one moment think that doctors, lawyers, and clergymen, should be expelled from civil society, any more than intelligent practical physicians would advocate the expunction of the name of a medicinal agent of recognized utility from the supply-table of a hospital, because it had been converted, in the hands of ignorance, into an instrument of destruction.

A very important question, pertinent to this discussion, seems not to have been asked, but if so, has not been answered; and that is,—Have the cases of gangrene, reported to the Medical Bureau, been caused by the use of mercury, or the insalubrity of the season, or of the particular locality where they have occurred!

More than thirty years ago, whilst on duty at a military outpost, I had opportunities of seeing cases of humid gangrene, such as have been described under the names of gangrenous erosion of the cheeks, gangrenopsis, and by that acutest of observers, the late Dr. Parrish, of Philadelphia, as a "disease resembling the effects of mercury." This disease was recognized as the product of malaria, and was especially familiar to the physicians residing at Natchez, on the Mississippi. Cases of this kind occurred in 1836 and 1839 as far north as Detroit; since when, owing to a notable change in the diathesis of epidemic disease, none have been seen, of which I have any knowledge, north of the Ohio River. So far from having been caused by the misuse of calomel, it was most successfully treated by heroic doses of this ostracised article.

I think it now quite reasonable to suppose that the approach of a similar morbid cycle co-operating with the exposures of a hazardous service, may have brought back the long lost cases of gangrenous erosion of the cheek. One reason for such a belief is derived from the condition of the sick found in the hospitals at Evansville, Ind., Paducah, Ken., Mound City and Cairo, Ill., and Memphis, Tennessee, where I saw in February last, instead of the destructive marks of excessive mercurialization, what I considered evidences of too cautious a use of mercurials in the early stages of the diarrhœas associated with or dependent upon hepatic torpor.

Yours, etc., Z. P.

Detroit, July, 1863.

